# Feature Engineering for Drug Name Recognition in Biomedical Texts: Feature Conjunction and Feature Selection

**DOI:** 10.1155/2015/913489

**Published:** 2015-03-12

**Authors:** Shengyu Liu, Buzhou Tang, Qingcai Chen, Xiaolong Wang, Xiaoming Fan

**Affiliations:** Key Laboratory of Network Oriented Intelligent Computation, Harbin Institute of Technology Shenzhen Graduate School, Shenzhen 518055, China

## Abstract

Drug name recognition (DNR) is a critical step for drug information extraction. Machine learning-based methods have been widely used for DNR with various types of features such as part-of-speech, word shape, and dictionary feature. Features used in current machine learning-based methods are usually singleton features which may be due to explosive features and a large number of noisy features when singleton features are combined into conjunction features. However, singleton features that can only capture one linguistic characteristic of a word are not sufficient to describe the information for DNR when multiple characteristics should be considered. In this study, we explore feature conjunction and feature selection for DNR, which have never been reported. We intuitively select 8 types of singleton features and combine them into conjunction features in two ways. Then, Chi-square, mutual information, and information gain are used to mine effective features. Experimental results show that feature conjunction and feature selection can improve the performance of the DNR system with a moderate number of features and our DNR system significantly outperforms the best system in the DDIExtraction 2013 challenge.

## 1. Introduction

Drug name recognition (DNR), which recognizes pharmacological substances from biomedical texts and classifies them into predefined categories, is an essential prerequisite step for drug information extraction such as drug-drug interactions [1]. Compared with other named entity recognition (NER) tasks, such as person, organization, and location name recognition in newswire domain [2] and gene name recognition [3] and disease name recognition [4] in biomedical domain, DNR has its own challenges. Firstly, drug names may contain a number of symbols mixed with common words, for example, “N-[N-(3, 5-difluorophenacetyl)-L-alanyl]-S-phenylglycine t-butyl ester.” Secondly, the ways of naming drugs vary greatly. For example, the drug “valdecoxib” has the brand name “Bextra,” while its systematic International Union of Pure and Applied Chemistry (IUPAC) name is “4-(5-methyl-3-phenylisoxazol-4-yl)benzenesulfonamide.” Thirdly, due to the ambiguity of some pharmacological terms, it is not trivial to determine whether substances should be drugs or not. For example, “insulin” is a hormone produced by the pancreas, but it can also be synthesized artificially and used as drug to treat diabetes.

Many efforts have been devoted to DNR, including several challenges such as DDIExtraction 2013 [5]. Methods developed for DNR mainly fall into three categories: ontology-based [1], dictionary-based [6], and machine learning-based [7] methods. Ontology-based methods map units of texts into domain-specific concepts and then combine the concepts into drug names. Dictionary-based methods identify drug names in biomedical texts by using lists of terms in drug dictionaries. Machine learning-based methods build machine learning models based on labeled corpora to identify drug names. Machine learning-based methods outperform the other two categories of methods when a large corpus is available.

Because of the lack of labeled corpora, early studies on DNR are mainly based on ontologies and dictionaries. Segura-Bedmar et al. [1] proposed an ontology-based method for DNR in biomedical texts. Firstly, the method maps biomedical texts to the Unified Medical Language System (UMLS) concepts by the UMLS MetaMap Transfer (MMTx) program [8]. Then nomenclature rules recommended by the World Health Organization (WHO) International Nonproprietary Names (INNs) Program are used to filter drugs from all the concepts. Sanchez-Cisneros et al. [6] presented a DNR system that integrates results of an ontology-based and a dictionary-based method by different voting systems.

To promote the research on drug information extraction, MAVIR research network and University Carlos III of Madrid in Spain organized two challenges successively: DDIExtraction 2011 and DDIExtraction 2013. Both of the two challenges provide labeled corpora that can be used for machine learning-based DNR. The DDIExtraction 2011 challenge [9] focuses on the extraction of drug-drug interactions from biomedical texts; therefore, only mentions of drugs are annotated in the DDIExtraction 2011 corpus. Based on the DDIExtraction 2011 corpus, He et al. [7] presented a machine learning-based system for DNR. In their system, a drug name dictionary is constructed and incorporated into a conditional random fields- (CRF-) based method. The DDIExtraction 2013 challenge [5] is also designed to address the extraction of drug-drug interactions, but DNR is presented as a separate subtask. Both mentions and types of drugs are annotated in the DDIExtraction 2013 corpus. Six teams participated in the DNR subtask of the DDIExtraction 2013 challenge. Methods used for the DNR subtask can be divided into two categories: dictionary-based and machine learning-based methods. A machine learning-based method achieved the best performance.

Recently, the Critical Assessment of Information Extraction systems in Biology (BioCreAtIvE) IV launched the chemical compound and drug name recognition (CHEMDNER) task [10, 11]. The CHEMDNER task contains two subtasks: chemical entity mention recognition (CEM) and chemical document indexing (CDI). However, the CEM subtask focuses on identifying not only drugs but also chemical compounds. The top-ranked systems in the CEM subtask are also based on machine learning-based algorithms [12–15].

Machine learning algorithms and features are two key aspects of machine learning-based methods. Many machine learning algorithms such as CRF [7] and support vector machines (SVM) [16] have been used for DNR. CRF is the most reliable one with the highest performance [5]. Various types of features such as part-of-speech (POS), word shape, and dictionary feature have been used in machine learning-based methods. These features are usually used alone. We call them singleton features. Singleton features capture only one linguistic characteristic of a word and they are insufficient in cases where multiple linguistic characteristics of a word should be considered. Conjunction features (i.e., combinations of singleton features) may contain new meaningful information beyond singleton features. A number of studies have shown that proper conjunction features are beneficial to machine learning-based NER systems. For example, Tang et al. [17] generated conjunction features by combining words and POS tags within a context window to improve their machine learning-based clinical NER system. Tsai et al. [18] combined any two words in a context window to generate word conjunction features and the word conjunction features improved the performance of the machine learning-based biomedical NER system. All of them use only conjunction features based on one or two kinds of singleton features. In this study, we investigate the effectiveness of conjunction features based on multiple kinds of singleton features to machine learning-based DNR systems. It is the first time to study conjunction features based on multiple kinds of singleton features for NER, and it is the first time to use conjunction features in machine learning-based DNR systems. The main challenge of using conjunction features is, which features should be used? It is not feasible to incorporate any conjunctions of singleton features into the machine learning model due to the extremely large feature set with millions of features and high computing cost. Moreover, improper conjunctions of features may introduce noises and result in performance decrease. A possible solution to remove improper features is feature selection.

In this study, we manually select 8 types of singleton features to generate conjunction features in two ways: (i) combining two features of the same type in the context window and (ii) combining two types of features in the context window. To remove improper conjunction features, we apply three popular feature selection methods, Chi-square, mutual information, and information gain to all features. Experimental results on the DDIExtraction 2013 corpus show that the combination of feature conjunction and feature selection is beneficial to DNR. The CRF-based DNR system achieves an *F*-score of 78.37% when only singleton features are used. *F*-score is improved by 0.99% when feature conjunction and feature selection are subsequently performed. Finally, the CRF-based DNR system achieves an *F*-score of 79.36%, which outperforms the best system in the DDIExtraction 2013 challenge by 7.86%.

## 2. Methods

### 2.1. Drug Name Recognition

DNR is usually formalized as a sequence labeling problem, where each word in a sentence is labeled with a tag that denotes whether a word is part of a drug name and its position in a drug name.

*BIO* and* BILOU* are the most two popular tagging schemes used for NER. In the* BIO* tagging scheme,* BIO*, respectively, represent that a token is at the beginning (*B*) of an entity, inside (*I*) of an entity, and outside of an entity (*O*). In the* BILOU* tagging scheme,* BILOU*, respectively, represent that a token is at the beginning (*B*) of an entity, inside (*I*) of an entity, last token of an entity (*L*), and outside of an entity (*O*) and is an unit-length entity (*U*). Compared with the* BIO* tagging scheme,* BILOU* are more expressive and can capture more fine-grained distinctions of entity components. Some previous studies [15, 17, 19] have also shown that* BILOU* outperform* BIO* on NER tasks in different fields. Following them, we adopt* BILOU* to label drug names in this study. As four types of drugs are defined in the DDIExtraction 2013 challenge, “*drug,*” “*brand,*” “*group*,” and “*no-human*,” 17 tags (*B*-drug, *I*-drug, *L*-drug, *U*-drug, *B*-brand, *I*-brand, *L*-brand, *U*-brand, *B*-group, *I*-group, *L*-group, *U*-group, *B*-no-human, *I*-no-human, *L*-no-human, *U*-no-human and *O*) are actually used in our DNR system.

CRF is a typical sequence labeling algorithm and has been demonstrated to be superior to other machine learning methods for NER. CRF-based method achieved the best performance on the DNR subtask of DDIExtraction 2013 challenge [5]. Moreover, CRF was also utilized by highly ranked systems on the medical concept extraction task of i2b2 2010 [20], bio-entity recognition task of JNLPBA [21], and gene mention finding task of BioCreAtIve [22]. Therefore, we use CRF in our DNR system. An open source implementation of CRF, CRFsuite (http://www.chokkan.org/software/crfsuite/), is used.

### 2.2. Singleton Features

Singleton features used for DNR in this paper are as follows.

*Word Feature.* The word feature is the word itself. 

*POS Feature*. POS type is generated by the GENIA (http://www.nactem.ac.uk/tsujii/GENIA/tagger/) toolkit for a word.

*Chunk Feature*. Chunk information is generated by the GENIA toolkit for a word. 

*Orthographical Feature*. Words are classified into four classes {“All-capitalized,” “Is-capitalized,” “All-digits,” and “Alphanumeric”} based on regular expressions. The class label is used as a word's orthographical feature. In addition, {“Y”, “N”} are used to denote whether a word contains a hyphen or not. 

*Affix Feature*. Prefixes and suffixes are of the length of 3, 4, and 5. 

*Word Shape Feature.* Similar to [27], two types of word shapes “generalized word class” and “brief word class” are used. The “generalized word class” maps any uppercase letter, lowercase letter, digit, and other characters in a word to “X,” “x,” “0,” and “O,” respectively, while the “brief word class” maps consecutive uppercase letters, lowercase letters, digits, and other characters to “X,” “x,” “0,” and “O,” respectively. For example, the word shapes of “Aspirin1+” are “Xxxxxxx0O” and “Xx0O.”

In addition to the above features that are commonly used for NER, dictionary features are also widely used in DNR systems [23, 24]. Three drug dictionaries are used to generate dictionary features in the way similar to [16], which denotes whether a word appears in a dictionary by {“Y”, “N”}. Dictionaries used in this paper are described as follows. 

*DrugBank.* DrugBank [28] contains 6825 drug entries including 1541 FDA-approved small molecule drugs, 150 FDA-approved biotech (protein/peptide) drugs, 86 nutraceuticals, and 5082 experimental drugs (http://www.drugbank.ca/downloads). 

*Drugs@FDA.* Drugs@FDA (http://www.fda.gov/Drugs/InformationOnDrugs/ucm079750.htm) is a database provided by U.S. Food and Drug Administration. It contains information about FDA-approved drug names, generic prescription, over-the-counter human drugs, and biological therapeutic products. Totally, 8391 drug names are extracted from the* Drugname* and* Activeingred* fields of Drugs@FDA.

*Jochem*. Jochem [29] is a joint chemical dictionary. 1527751 concepts are extracted from Jochem.

Moreover, word embeddings feature that can capture semantic relations among words is also used. 

*Word Embeddings Feature*. Word embeddings learning algorithms can induce dense, real-valued vector representations (i.e., word embeddings) from large-scale unstructured texts for words. We use the skip-gram model proposed in [30] to learn word embeddings on the article abstracts in 2013 version of MEDLINE (http://www.nlm.nih.gov/databases/journal.html). Following previous works, we set the dimension of word embeddings to 50 and the word2vec tool (https://code.google.com/p/word2vec/) is used as an implement of the skip-gram model. After inducing word embeddings for words, words are clustered into different semantic classes by *k*-means clustering algorithm. The semantic class that a word belonged to is used as its word embeddings feature. The optimal number of semantic classes is selected from {100,200,300,…, 1000} via 10-fold cross-validation on the training set of the DDIExtraction 2013 challenge and 400 is determined as the optimal number.

### 2.3. Feature Conjunction

Conjunction features are directly generated by combining singleton features together. The templates used to generate singleton features are shown in Table 1, where *f*_*i*_[*j*] is the *i*th singleton feature at the *j*th position in the context window for a current word *w*_0_. For example, *f*_1_[−1] is a singleton feature of “the previous word of *w*_0_.” A template to generate conjunction features can be denoted by a *k*-tuple as *f*_*i*_1__[*j*_1_]_*f*_*i*_2__[*j*_2_]_ ⋯ _*f*_*i*_*m*__[*j*_*m*_]_ ⋯ _*f*_*i*_*k*__[*j*_*k*_], where 1 ≤ *i*_*m*_ ≤ 16, −*n* ≤ *j*_*m*_ ≤ *n*, and 2*n* + 1 is the context window size of *w*_0_. The number of conjunction features will explosively increase when increasing the context window size and the dimension of conjunction feature tuples. Following the previous studies [17, 18], we only consider 2-tuple conjunction features with context window size of 5.

When adding the conjunction features into machine learning-based DNR systems, it is important to keep effective conjunction features and avoid noisy ones. In this study, the effectiveness of conjunction features is determined by manually checking some samples in the training set. Take the conjunction of chunk feature and dictionary feature for example. On one hand, drug names are usually located in noun phrases, one type of chunks. However, only a small part of noun phrases contains drug names. On the other hand, words in drug dictionaries are of certain probabilities in drug names. When a word appears in a noun phrase and a drug dictionary, it is very likely in a drug name. These features are helpful to solve ambiguous pharmacological terms mentioned before. On the contrary, the conjunction of affixes of words is noisy. Consider the target words “*interleukin-2,*” “*interferon-alfa,*” “*intensive,*”* and* “*intraluminal*” in the following four sentences. The prefixes of length of 3 of the four target words are all “*int-*” and that of their following words are all “*con-*”. The four target words have the same conjunction prefix feature “*int-_con-*”. However, “*interleukin-2*” and “*interferon-alfa*” are drug names, and “*intensive*” and “*intraluminal*” are not.

- Delayed adverse reactions to iodinated contrast media: a review of the literature revealed that 12.6% (range 11–28%) of 501 patients treated with various* interleukin-2* containing regimens who were subsequently administered radiographic iodinated contrast media experienced acute, atypical adverse reactions.
- Myocardial injury, myocardial infarction, myocarditis, ventricular hypokinesia, and severe rhabdomyolysis appear to be increased in patients receiving PROLEUKIN and* interferon-alfa* concurrently.
- There is now strong evidence that* intensive* control of blood glucose can significantly reduce and retard the microvascular complications of retinopathy, nephropathy, and neuropathy.
- The effects of alosetron on monoamine oxidases and on intestinal first pass secondary to high* intraluminal* concentrations have not been examined.

Finally, conjunction features generated from 8 out of 16 singleton feature templates (*f*_1_–*f*_8_) in Table 1 are proved to be effective. Actually, the 2-tuple conjunction features used in this paper are extracted in the following two ways.

(1) Two singleton features of the same type in the context window are combined. The corresponding conjunction feature template set *S*_1_ is defined as

(1) $$\begin{array}{c} S_{1}=\left\{f_{i}\left[m\right]\text{\_}f_{i}\left[m+1\right]\;|\;i\;=\;1,2,3;\;-2\leq m\leq 1\right\}. \end{array}$$

*S*_1_ extracts bigrams of *f*_1_, *f*_2_, and *f*_3_ in the context window. For the target word *w*_0_, 12 conjunction features are extracted by *S*_1_.

(2) Different types of singleton features of the target word are combined. The corresponding conjunction feature template set *S*_2_ is defined as

(2) S2={fi0_fj0 ∣ i  <  j;  2  ≤  i,  j≤8}∪{f10_f20,f10_f30}.

In *S*_2_, *f*_1_ of the target word is only combined with *f*_2_ and *f*_3_ of it. For *f*_2_–*f*_8_, any two of them are combined. For each target word *w*_0_, 23 conjunction features are extracted by *S*_2_.

**Figure 1:**
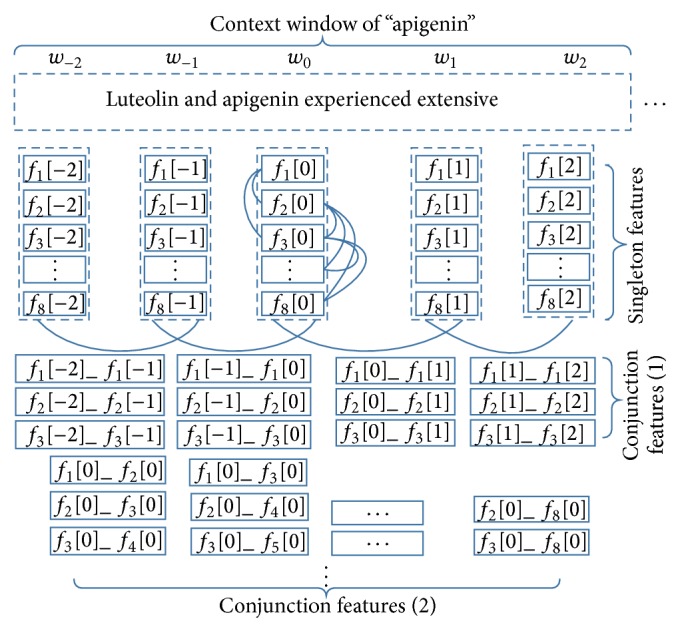
Conjunction features for “apigenin.”

Figure 1 shows the conjunction features for “apigenin” in “luteolin and apigenin experienced extensive …” in the above two ways.

### 2.4. Feature Selection

Three commonly used feature selection methods in the text processing field, Chi-square [31], mutual information [32], and information gain [33], are used for feature selection for further improvement. Fundamental concepts and the usage of Chi-square, mutual information, and information gain are presented in below parts of this section, which make use of the symbol system in [34].

#### 2.4.1. Chi-Square

Chi-square is usually used to test the independence of two events *A* and *B*. For feature selection in DNR, the two events *A* and *B* are, respectively, defined as occurrence of a feature and occurrence of a tag, that is, tags in {*B*, *I*, *L*, *O*, *U*}. For feature *f* and tag *t*, Chi-square value is defined as

(3) $$\begin{array}{c} \text{Chi}\left(f,t\right)=\underset{e_{f}\in \left\{0,1\right\}}{\sum }\;\underset{e_{t}\in \left\{0,1\right\}}{\sum }\frac{{\left(N_{e_{f}e_{t}}-E_{e_{f}e_{t}}\right)}^{2}}{E_{e_{f}e_{t}}}, \end{array}$$

where *e*_*f*_ = 1 denotes that current word has feature *f* and *e*_*f*_ = 0 denotes that current word does not have feature *f*; *e*_*t*_ = 1 denotes that tag of current word is *t* and *e*_*t*_ = 0 denotes that tag of current word is not *t*. *N* is the observed frequency in the training corpus and *E* is the expected frequency conditioned on the independence between the occurrence of *f* and the occurrence of *t*. For example, *N*_11_ is the observed frequency of words that have feature *f* and tag *t* in the training set. *E*_11_ is the expected frequency of words that have feature *f* and tag *t* assuming that the occurrence of *f* and the occurrence of *t* are independent. The higher the Chi-square value of feature *f* is, the more important the feature *f* is. The importance measure *I*(*f*) of feature *f* derived from Chi-square is defined as

(4) $$\begin{array}{c} I\left(f\right)=\underset{t\in \left\{B,I,L,O,U\right\}}{\max}\left\{\text{Chi}\left(f,t\right)\right\}. \end{array}$$

#### 2.4.2. Mutual Information

For feature selection in DNR, mutual information measures how much information the presence or absence of feature *f* contributes to making the correct tagging decision on tag *t*. Mutual information between *f* and *t* is defined as

(5) MIf,t=∑ef∈0,1 ∑et∈0,1PF=ef,C=etlog2PF=ef,C=etPF=efPC=et,

where *e*_*f*_ and *e*_*t*_ are the same as that in (3). *F* is a random variable that takes values *e*_*f*_ = 1 (current word has feature *f*) and *e*_*f*_ = 0 (current word does not have feature *f*), and *C* is a random variable that takes values *e*_*t*_ = 1 (tag of current word is *t*) and *e*_*t*_ = 0 (tag of current word is not *t*). The importance measure *I*(*f*) of feature *f* derived from mutual information is defined as

(6) $$\begin{array}{c} I\left(f\right)=\underset{t\in \left\{B,I,L,O,U\right\}}{\max}\left\{\text{MI}\left(f,t\right)\right\}. \end{array}$$

#### 2.4.3. Information Gain

Information gain measures how much information the presence or absence of feature *f* contributes to the DNR system as a whole. Information gain of feature *f* to the DNR system is defined as

(7) IGf=H(T)−H(T ∣ ef)=−∑T∈{B,I,L,O,U}P(T)log2P(T) +P(ef=1)∑T∈B,I,L,O,UP(T ∣ ef=1)log2P(T ∣ ef=1) +P(ef=0)∑T∈{B,I,L,O,U}P(T ∣ ef=0)log2P(T ∣ ef=0),

where *e*_*f*_ is the same as that in (3), *H* denotes information entropy, and *T* is a random that takes values in {*B*, *I*, *L*, *O*, *U*}. The importance measure *I*(*f*) of feature *f* derived from information gain is defined as

(8) $$\begin{array}{c} I\left(f\right)=\text{IG}\left(f\right). \end{array}$$

## 3. Results

To investigate the effectiveness of feature conjunction and feature selection on DNR, we start with the system that uses only singleton features and then feature conjunction and feature selection are successively performed. All experiments are conducted on the DDIExtraction 2013 corpus. Each CRF model uses default parameters except regularization coefficient. The optimal regularization coefficient is selected from {0.5,0.6,…, 1.5} via 10-fold cross validation on the training set of the DDIExtraction 2013 challenge.

### 3.1. Data Set

The DDIExtraction 2013 corpus consists of 826 documents, which come from two sources: DrugBank and MEDLINE. 15450 drug names are annotated and classified into four types:* drug*,* group*,* brand*, and* no-human *[5]. The corpus is split into two parts: a training set and a test set. Both of them contain a part of documents from DrugBank and MEDLINE. Training set of the corpus is used for system development, while test set is used for system evaluation.  Table 2 gives statistics of the DDIExtraction 2013 corpus.

### 3.2. Evaluation Metrics

The DDIExtraction 2013 challenge provides four criteria for evaluation of the DNR systems. precision (*P*), recall (*R*), and *F*-score (*F*1) are used to evaluate the performances of the DNR systems under the criteria. The four criteria are as follows.

- Strict matching: a predicted drug name is correct when and only when both the boundary and type of it exactly match with a gold one.
- Exact boundary matching: a predicted drug name is correct when the boundary of it matches with a gold one regardless of its type types.
- Type matching: a predicted drug name is correct when it overlaps a gold one of the same type.
- Partial boundary matching: a predicted drug name is correct when it overlaps a gold one regardless of its type.

In the DDIExtraction 2013 challenge, strict matching is used as the primal criterion.

### 3.3. Experimental Results

Table 3 shows the experimental results under strict matching criterion. *F*_*s*_ denotes all singleton features. *F*_*c*_ denotes the proposed conjunction features. *F*_*o*_ denotes the optimal feature subset determined by the feature selection method, information gain.

It can be seen that both feature conjunction and feature selection are beneficial to DNR. When only singleton features are used, the DNR system achieves an *F*1 of 78.37%. A 0.2% improvement of *F*1 is achieved by the proposed conjunction features. *F*1 is further improved by 0.79% after eliminating negative effects of the noisy features by information gain. Finally, an improvement of *F*1 is achieved by 0.99% (from 78.37% to 79.36%) by using feature conjunction and feature selection in combination.

### 3.4. Comparisons between Our System and All Systems in the DDIExtraction 2013 Challenge

To further investigate our DNR system, we compare our best system with all participating systems in the DDIExtraction 2013 challenge. The comparison is shown in Table 4. Our system outperforms the best performing system in the DDIExtraction 2013 challenge, WBI, by an *F*1 of 7.86%. The detailed comparisons between WBI and our system are shown in Table 5, where the overall performances under the four criteria and the performances of each type of drugs under the strict matching criterion are listed.

Our system outperforms WBI when types of drugs are considered. The differences of *F*1 under the strict matching and type matching criteria are 7.86% and 7.29%, respectively. The differences of *F*1 are small if types of drugs are not considered. For exact matching and partial matching, the differences of *F*1 are 0.55% and 0.22%, respectively. For each type of drugs, our system achieves better performance than WBI. The improvements of *F*1 range from 1.04% (for* no-human*) to 13.79% (for* brand*).

### 3.5. Effects of Feature Selection on DNR

**Figure 2:**
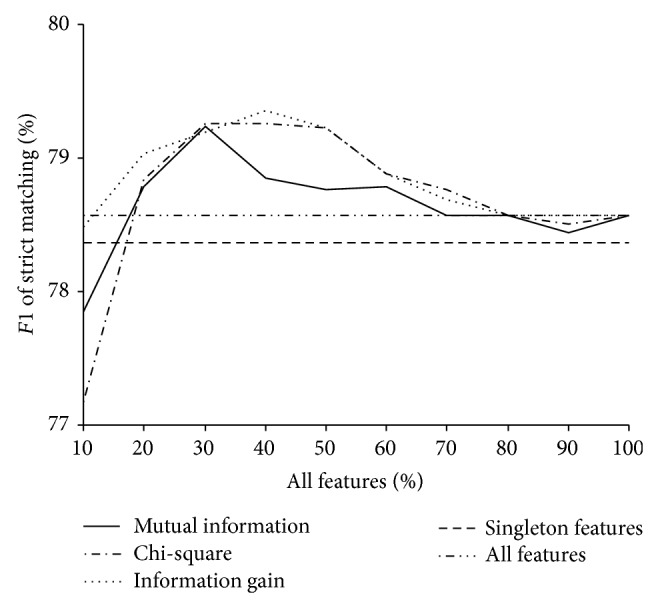
Performance curves of DNR systems with different percentages of all features.

To investigate the effects of feature selection on DNR, we compare the performances of DNR systems when different feature selection methods are used.  Figure 2 shows the performance curves of DNR systems when using different percentages (10%, 20%, 30%,…, 100%) of all features selected by Chi-square, mutual information, and information gain. When top 30% of features selected by Chi-square are used, the system achieves the best performance with an *F*1 of 79.26%. For mutual information, when top 30% of features are used, the system achieves the best performance with an *F*1 of 79.24%. For information gain, when top 40% of features are selected, the system performs best and achieves an *F*1 of 79.36%.

Moreover, when more than 30% (for Chi-square and mutual information) or 40% (for information gain) of features are used, performances of the systems decline gradually. When fewer than 30% (for Chi-square and mutual information) or 40% (for information gain) of features are used, performances of the systems decline sharply. This demonstrates that features selected by the feature selection methods are effective for DNR.

It can also be observed in Figure 2 that three feature selection methods are comparable with each other. The performance differences of the systems are small when different feature selection methods are used. Moreover, the sizes of the optimal feature subsets determined by three feature selection methods are close. The optimal feature subsets determined by Chi-square, mutual information, and information gain contain 30%, 30%, and 40% of all features, respectively.

### 3.6. Effects of Singleton Features for DNR

As seen in Table 1, surface level features used in our system include word feature (*f*_1_), orthographical feature (*f*_4_), affix feature (*f*_9_–*f*_14_), and word shape (*f*_15_-*f*_16_). Besides surface level features, we made use of some external resources to generate features. The GENIA toolkit is used to generate syntactic features (*f*_2_-*f*_3_). Three drug dictionaries are used to generate dictionary features (*f*_5_–*f*_7_). MEDLINE abstracts are used to induce unsupervised word embeddings feature (*f*_8_). To investigate the effectiveness of surface level features and features based on external resources, we compare the performances of DNR systems using different features.  Table 6 shows the experimental results under strict matching criterion, where *F*_sur_, *F*_syn_, *F*_dic_, and *F*_emb_ denote surface level features, syntactic features, dictionary features, and unsupervised word embeddings feature, respectively. It can be seen that features based on three different external resources are all beneficial to DNR. When *F*_syn_, *F*_dic_, and *F*_emb_ are added to *F*_sur_, respectively, *F*1 is improved by 1.47%, 5.50%, and 3.52%, respectively. Furthermore, the external features are accumulative. When any two external features are added to *F*_sur_ simultaneously, performance of the system outperforms that of the system using one single external feature. When all external features are added, the system achieves the best performance.

## 4. Discussion

In this paper, we investigate the effectiveness of feature conjunction and feature selection on DNR. When only singleton features are used, our baseline system achieves an *F*1 of 78.37%, which outperforms the best system in the DDIExtraction 2013 challenge (i.e., WBI) by an *F*1 of 6.87%. When the proposed conjunction features are added, our system achieves better performance with an *F*1 of 78.57%. After feature selection, our system is further improved with an *F*1 of 79.36%.

Compared with the top-ranked systems of the DDIExtraction 2013 challenge, WBI and LASIGE, also based on CRF, our system shows better performance mainly because of different features, such as drug dictionary features, and additional features, such as unsupervised word embedding features and conjunction features. The dictionaries used in WBI and LASIGE consist of a large number of chemical compounds with a small part of drugs, whereas the dictionaries used in our system completely consist of drugs. It is easy to understand that the dictionaries used in our system are more helpful than those used in WBI and LASIGE for DNR. In the case of unsupervised word embedding features and conjunction features, they have been proved to be very beneficial to DNR as shown in Tables 6 and 3, respectively.

Although our baseline system, which uses various types of singleton features, significantly outperforms the state-of-the-art system, its performance can still be improved by feature conjunction and feature selection. To our best knowledge, it is the first time to investigate the effects of feature conjunction and feature selection on DNR. The combination of feature conjunction and feature selection improves *F*1 of our DNR system by 0.99% (from 78.37% to 79.36%). It is easy to understand that feature conjunction and feature selection can improve the performance of the DNR system. Because feature conjunction generates mounts of conjunction features which can capture multiple characteristics of the words and thus accurately represent the context of drug names and feature selection can remove noisy features to eliminate negative effects of them, for this reason, the precision is improved by 3.62% (from 84.75% to 88.37%) when feature conjunction and feature selection are performed as shown in Table 3.

However, no conjunctions of singleton features are beneficial to DNR. Some improper conjunctions of singleton features can decrease the performances of DNR systems. For example, when the bigrams of *f*_15_ (generalized word class) in the context window (i.e., features extracted by *f*_15_[−2]_*f*_15_[−1], *f*_15_[−1]_*f*_15_[0], *f*_15_[0]_*f*_15_[1], and *f*_15_[1]_*f*_15_[2]) are added to the baseline DNR system, the performance of the system drops from 78.37% to 76.86%. This is mainly because there are no inherent relations between generalized word classes of two consecutive words. Therefore, we manually select 8 out of 16 singleton feature templates, between which there are inherent relations intuitively, to generate conjunction feature templates.

Chi-square, mutual information, and information gain are used to eliminate negative effects of noisy features. As shown in Figure 2, each of the three feature selection methods is beneficial to DNR and the performances of them are comparable with each other. Finally, the top 40% of all features selected by information gain are used as the optimal feature subset for our DNR system. It is also important to note that the number of features is reduced from 294782 to 117913 by information gain as shown in Table 3. Therefore, feature selection not only can improve the performance of the DNR system, but also can make the DNR system more efficient with fewer features.

Although the performance of our DNR system is better than that of WBI, it is not good enough. The performance for the “*no-human*” type is still poor. This is mainly because four types of drugs in the corpus are extremely imbalanced. Drug names of the “*no-human*” type account for only 4% of all drug names in the training set, while drug names of the “*drug*” type account for about 63%. We will explore solving the data imbalance problem in the future.

## 5. Conclusions

In this paper, we investigate the effectiveness of feature conjunction and feature selection on DNR. Experiments on the DDIExtraction 2013 corpus show that the combination of feature conjunction and feature selection is beneficial to DNR. For future work, it is worth investigating other ways of combining singleton features and other feature selection methods for DNR.

## Figures and Tables

**Table 1:** Singleton feature templates.

Number	Feature template
*f*_1_	Word feature
*f*_2_	POS
*f*_3_	Chunk
*f*_4_	Orthographical feature
*f*_5_	DrugBank
*f*_6_	FDA
*f*_7_	Jochem
*f*_8_	Word embeddings feature
*f*_9_	Prefix of length of 3
*f*_10_	Prefix of length of 4
*f*_11_	Prefix of length of 5
*f*_12_	Suffix of length of 3
*f*_13_	Suffix of length of 4
*f*_14_	Suffix of length of 5
*f*_15_	Word shape (generalized word class)
*f*_16_	Word shape (brief word class)

**Table 2:** Statistics of the DDIExtraction 2013 corpus.

	DrugBank			MEDLINE		
	Training	Test	Total	Training	Test	Total
Documents	572	54	626	142	58	200
Sentences	5675	145	5820	1301	520	1821
Drug	8197	180	8377	1228	171	1399
Group	3206	65	3271	193	90	283
Brand	1423	53	1476	14	6	20
No-human	103	5	108	401	115	516

**Table 3:** Experimental results of the DNR systems on the DDIExtraction 2013 corpus under strict matching criterion (%).

Feature	Feature number	*P*	*R*	*F*1
*F*_*s*_	43935	84.75	72.89	78.37
*F*_*s*_ + *F*_*c*_	294782	86.64	71.87	78.57
*F*_*o*_	117913	88.37	72.01	**79.36**

**Table 4:** Comparisons between our system and all systems in the DDIExtraction 2013 challenge (%).

Method	Strict		
	*P*	*R*	*F*
Our system	88.37	72.01	**79.36**
WBI [23]	73.40	69.80	71.50
NLM_LHC	73.20	67.90	70.40
LASIGE [24]	69.60	62.10	65.60
UTurku [16]	73.70	57.90	64.80
UC3M [25]	51.70	54.20	52.90
UMCC_DLSI-(DDI) [26]	19.50	46.50	27.50

**Table 5:** Detailed comparisons between WBI and our system (%).

	WBI			Our system			Δ*F*1
	*P*	*R*	*F*1				
Strict	73.40	69.80	71.50	88.37	72.01	79.36	+7.86
Exact	85.50	81.30	83.30	93.38	76.09	83.85	+0.55
Type	76.70	73.00	74.80	91.41	74.49	82.09	+7.29
Partial	87.70	83.50	85.60	95.08	77.48	85.38	−0.22

Drug (strict)	73.60	85.20	79.00	93.35	88.03	90.61	+11.61
Brand (strict)	81.00	86.40	83.60	100.0	94.92	97.39	+13.79
Group (strict)	79.20	76.10	77.60	90.15	76.77	82.92	+5.32
No-human (strict)	31.40	9.10	14.10	90.91	8.26	15.14	+1.04

**Table 6:** Experimental results of the DNR systems using different features on the DDIExtraction 2013 corpus under strict matching criterion (%).

Feature	*P*	*R*	*F*1
*F*_sur_	81.04	63.56	71.24
*F*_sur_ + *F*_syn_	78.41	67.78	72.71
*F*_sur_ + *F*_dic_	86.05	69.24	76.74
*F*_sur_ + *F*_emb_	84.69	66.91	74.76
*F*_sur_ + *F*_syn_ + *F*_dic_	83.84	71.87	77.39
*F*_sur_ + *F*_syn_ + *F*_emb_	82.70	69.68	75.63
*F*_sur_ + *F*_dic_ + *F*_emb_	85.56	69.97	76.98
*F*_sur_ + *F*_syn_ + *F*_dic_ + *F*_emb_	84.75	72.89	78.37
